# Epilepsy-IEDs: An automated machine learning model for detecting interictal epileptiform discharges from scalp electroencephalograms

**DOI:** 10.1016/j.isci.2026.116408

**Published:** 2026-06-22

**Authors:** Ran Ao, Ping Zhan, Guojing Wang, Hongyun Liu, Weidong Wang

**Affiliations:** 1 Medical Innovation Research Division, Chinese PLA General Hospital, Beijing 100853, China; 2 Key Laboratory of Biomedical Engineering and Translational Medicine, Ministry of Industry and Information Technology, Beijing 100853, China

**Keywords:** Neuroscience, Sensory neuroscience, Techniques in neuroscience, Machine learning

## Abstract

Interictal epileptiform discharges (IEDs) are essential for epilepsy diagnosis, yet visual electroencephalogram (EEG) analysis remains subjective and laborious. To address this, we developed Epilepsy-IEDs, an automated machine learning model for IED detection. Trained on 141 scalp EEG recordings (2,597 IEDs and 4,633 non-IEDs), the model was evaluated using four algorithms, with a separate daytime analysis to control for sleep effects. The Extreme Gradient Boosting (XGBoost)-based model achieved the highest performance, with sensitivities of 84.6% (area under the curve [AUC] = 0.966) and 87.1% (AUC = 0.973) on the full and daytime datasets, respectively, and demonstrated robust generalization on held-out epilepsy patients (AUC = 0.878–0.890), with a specificity of 71.54% on a non-epilepsy cohort. A simplified 10-feature variant maintained strong performance (AUC = 0.959). The Epilepsy-IEDs model provides an accurate, interpretable tool for IED detection, with a streamlined version suitable for integration into clinical EEG workflows.

## Highlights

• Epilepsy-IEDs: an ML model for automated IED detection
• XGBoost achieves 84.6% sensitivity and 0.966 AUC
• A 10-feature variant maintains strong performance (AUC 0.959)
• SHAP analysis identifies key features for clinical interpretability

## Graphical abstract

**Figure:**
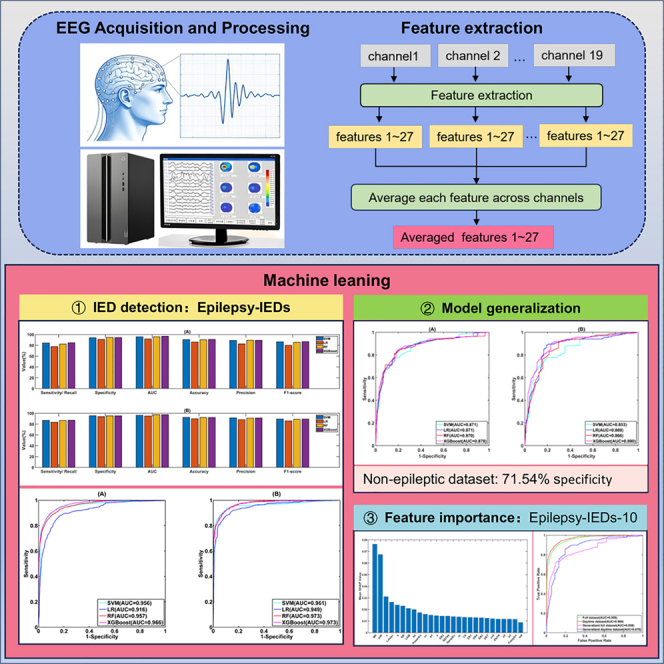

## Introduction

Epilepsy is one of the most common neurological disorders, characterized by aberrant neural network formation and hypersynchronous neuronal discharges in the brain.^1^ According to statistics, the number of people with epilepsy worldwide exceeds 70 million, with an annual incidence rate of 5/10,000.^2^ The scalp electroencephalogram (EEG) is widely explored as a fundamental medical test for diagnosis of epilepsy.^3^^,^^4^^,^^5^^,^^6^ However, clinical reliance on ictal events for diagnosis remains challenging due to their infrequent occurrence and variable presentation.^7^ As an alternative, interictal EEG analysis has emerged as a valuable diagnostic strategy. Approximately 50% of the routine EEG recordings in patients with epilepsy demonstrate interictal epileptiform discharges (IEDs), and a proportion increases to 80% in sleep recordings.^8^^,^^9^ IEDs refer to transient electrographic abnormalities characterized by sharp waves, spike waves, or spike-and-slow wave complexes that stand out against the background on an EEG, without obvious clinical manifestations of epileptic seizures. IEDs are of significant value for diagnosing epilepsy, differentiating types of epileptic seizures, identifying subtle seizures, and localizing epileptic foci.

The gold standard for IED detection is visual analysis by experts, which has some obvious shortcomings, including high dependence on subjective judgment, , time-consuming nature, proneness to errors, and difficulty in dealing with the analysis of large-scale data.^10^ These limitations have prompted researchers to explore automated detection methodologies to enhance clinical efficiency and diagnostic accuracy. As an important branch of artificial intelligence, machine learning (ML) has been widely used for IED identification by deciphering subtle electrographic patterns that may elude human observers. ML enhances the consistency and accuracy of diagnoses, which is crucial for formulating effective treatment plans. Furthermore, it standardizes the diagnostic process, ensuring that patients receive equitable care regardless of the expertise of the individual clinician. Table 1 presents a systematic overview of contemporary ML-based IED detection studies. Notwithstanding these advancements, several methodological limitations persist across the literature. Most studies use different datasets to train their models, which hinders the comparison of the results. Some models are only trained on very small datasets,^11^^,^^12^^,^^13^^,^^14^^,^^15^^,^^16^ which are not representative of IED diversity. Additionally, several studies do not provide the objective assessment values needed to compare with other methods.^11^^,^^17^ Moreover, there are cases where the training and testing sets are not independently partitioned, resulting in overlapping patients between the two sets.^12^^,^^15^^,^^18^^,^^19^ This may lead to overfitting during model training and inaccurate evaluation of the model’s generalization ability, ultimately undermining the reliability and validity of these findings.

**Table 1:** Studies employing ML for IED detection using EEG signal

Study	Patient count	Method	Performance
Horak et al.^11^	9	Template matching + SVM	Template matching led to higher agreement with experts when compared to template matching + SVM
Zacharaki et al.^12^	1	SVM	Sensitivity = 97%, specificity = 63%, FP rate = 0.1
Le Douget et al.^13^	17	Wavelet transform + RF	Sensitivity = 62%, precision = 26%
Thomas et al.^18^	50	*k*-Means, k-medoids, fuzzy C-means clustering, agglomerative clustering, affinity propagation, template matching	The affinity propagation-based template matching system led to the highest AUC = 0.953
Bagheri et al.^17^	93	SVM	The cascade method increases precision, decreasing FP detections
Le et al.^14^	19	DBN	Sensitivity = 87.35%, specificity = 97.89%
Thomas et al.^20^	93	CNN + SVM	AUC = 0.870, accuracy = 83.86%
Bagheri et al.^19^	93	SVM, KNN, and RF	Sensitivity = 97%, specificity = 78%
Wang et al.^15^	7	RF	ACC = 96%, sensitivity = 97%, specificity = 96%
Thomas et al.^21^	84	CNN	Sensitivity = 80.0%, AUC = 0.98, precision = 82%, F1-score = 0.80, FP rate = 0.2
Sabor et al.^22^	390	LSTM	Sensitivity = 95.3%, precision = 87.3%, FP rate = 4.5%
Chan et al.^23^	20	CNN and CNN + LSTM	Sensitivity = 83%, precision = 92%, F1-score = 0.87
Tong et al.^16^	8	Transformer	Sensitivity = 97.8%, accuracy = 99.8%
Current study	141	SVM, RF, LR, and XGBoost	Sensitivity = 84.48%, AUC = 0.967, specificity = 94.71%, accuracy = 90.73%, precision = 89.62%, F1-score = 0.867

SVM, support vector machine; RF, random forest; DBN, deep belief network; CNN, convolutional neural network; KNN, k-nearest neighbors; LSTM, long short-term memory network; LR, logistic regression; XGBoost, Extreme Gradient Boosting; FP, false-positive.

In recent years, deep learning (DL) architectures including convolutional neural network (CNN), long short-term memory network, deep belief network, and transformer have been increasingly used in IED detection and have demonstrated superior performance,^14^^,^^16^^,^^20^^,^^21^^,^^22^^,^^23^ particularly in analyzing large-scale datasets and complex EEG signal. DL has the advantages of automatic feature extraction and pattern recognition. However, it has high requirements for data volume and computing resources, and its interpretability is relatively poor. Comparatively, traditional ML methods highly depend on the quality of feature extraction. When dealing with complex and nonlinear EEG signals, the generalization ability of traditional ML may be insufficient. Nevertheless, traditional ML offers advantages over DL in scenarios with limited data or the need for model interpretability. These models are preferred for their simplicity, faster training and inference, and ease of optimization.

In this study, we proposed an ML model to detect IEDs for epileptic patients (Epilepsy-IEDs). Rather than developing an entirely new algorithmic architecture, this study contributes a systematic evaluation of four ML algorithms (support vector machine [SVM], logistic regression [LR], random forest [RF], and Extreme Gradient Boosting [XGBoost]) on a relatively large clinical dataset, with a focus on optimizing feature engineering strategies to achieve strong overall performance with balanced sensitivity and specificity. To assess model performance under conditions that better reflect routine clinical practice, we conducted a separate analysis on daytime EEG recordings to minimize sleep-related confounding. Additionally, we developed a simplified 10-feature variant (Epilepsy-IEDs-10) to enhance clinical feasibility without compromising detection performance.

## Results

### IED detection performance

**Figure 1:**
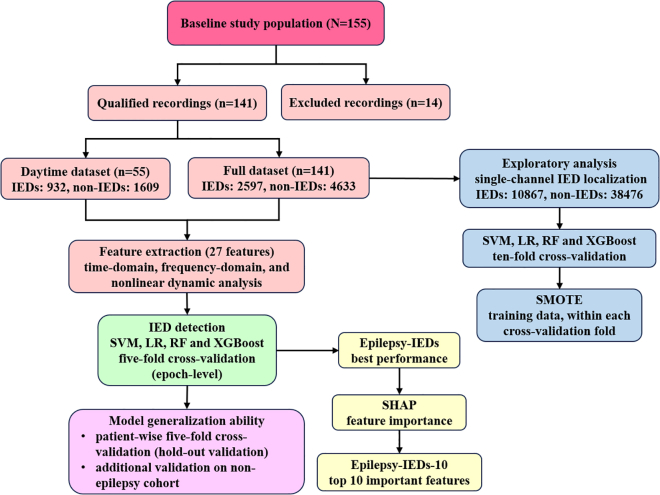
Flowchart of study population and overall analytical pipeline IED, interictal epileptiform discharges; SVM, support vector machine; LR, logistic regression; RF, random forest; XGBoost, Extreme Gradient Boosting; SHAP, Shapley Additive Explanations; SMOTE, synthetic minority oversampling.

The study population flow and analytical pipeline are illustrated in Figure 1. The performance metrics are defined in Table 2. The 5-fold cross-validation results of IED detection for the full dataset are presented in Table 3. All ML methods achieved robust performance, with area under the curve (AUC) ≥ 0.910 (range: 0.916–0.966), specificity ≥ 90% (range: 90.8%–94.5%), precision ≥ 82% (range: 82.6%–89.3%), and F1-score ≥ 0.800 (range: 0.800–0.868). The sensitivity (or recall) values were greater than 82% (range: 82.2%–84.6%) for SVM, RF, and XGBoost. RF demonstrated the highest specificity (94.5%; 95% confidence interval [CI], 93.8%–95.1%) and precision (89.3%; 95% CI, 88.0%–90.4%). XGBoost emerged as the top performance across metrics including sensitivity (84.6%; 95% CI, 83.2%–86.0%), AUC (0.966; 95% CI, 0.962–0.970), accuracy (90.8; 95% CI, 90.2%–91.5%), and F1-score (0.868; 95% CI, 0.858–0.878). LR underperformed other methods across all metrics, while RF and XGBoost exhibited complementary strengths in certain metrics.

**Table 2:** Performance metrics of ML for IED detection

Performance metrics	Measure	Description
Sensitivity/recall	$\frac{\text{TP}}{\text{TP}+\text{FN}}$	The proportion of correctly identified positive instances out of all actual positive instances
Specificity	$\frac{\text{TN}}{\text{TN}+\text{FP}}$	The proportion of correctly identified negative instances out of all actual negative instances
Accuracy	$\frac{\text{TP}+\text{TN}}{\text{TP}+\text{TN}+\text{FP}+\text{FN}}$	The ratio of correctly predicted instances to the total number of instances
AUC	–	The area under the ROC curve, which represents the model’s ability to discriminate between classes
Precision	$\frac{\text{TP}}{\text{TP}+\text{FP}}$	The proportion of true-positive predictions out of all positive predictions
F1-score	$\frac{2*\text{Precision}*\text{Recall}}{\;\text{Precision}+\text{Recall}\;}$	The harmonic mean of precision and recall, a balanced measure of precision and sensitivity

**Table 3:** IED detection performance for different ML methods using 5-fold cross-validation for the full dataset

Method	Sensitivity (%)	Specificity (%)	AUC (--)	Accuracy (%)	Precision (%)	F1-score (--)
SVM	84.2 (82.9, 85.6)	94.1 (93.4, 94.8)	0.956 (0.951, 0.961)	90.5 (89.8, 91.2)	88.9 (87.6, 90.1)	0.865 (0.855, 0.874)
LR	77.5 (76.0, 79.2)	90.8 (90.0,91.7)	0.916 (0.909, 0.923)	86.0 (85.4, 86.8)	82.6 (0.811, 0.841)	0.800 (0.787, 0.813)
RF	82.2 (80.6, 83.5)	94.5 (93.8, 95.1)	0.957 (0.953, 0.962)	90.2 (89.6, 90.9)	89.3 (88.0, 90.4)	0.855 (0.844, 0.865)
XGBoost	84.6 (83.2, 86.0)	94.3 (93.6, 95.0)	0.966 (0.962, 0.970)	90.8 (90.2, 91.5)	89.2 (87.9, 90.3)	0.868 (0.858, 0.878)

The data are reported as mean and 95% confidence interval.

RF, random forest; XGBoost, Extreme Gradient Boosting; CI, confidence interval.

**Figure 2:**
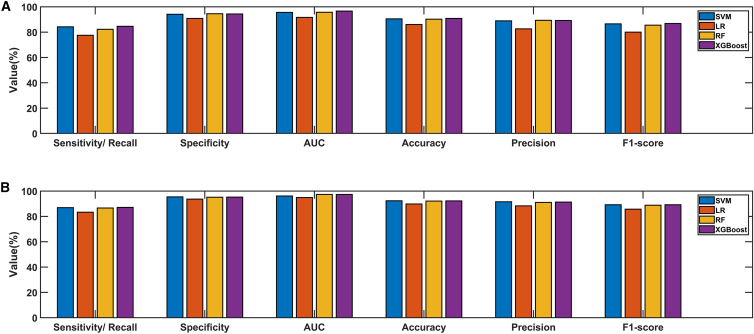
The IED detection performance for SVM, LR, RF, and XGBoost using 5-fold cross-validation (A) Full dataset: RF shows the best performance in terms of specificity, and precision. XGBoost shows the best performance in terms of sensitivity, AUC, accuracy, and F1-score. (B) Daytime dataset: SVM shows the best performance in terms of specificity, accuracy, precision, and F1-score. XGBoost shows the best performance in terms of sensitivity, AUC, and F1-score. RF and XGBoost exhibit superior performance in terms of AUC. IED, interictal epileptiform discharges. SVM, support vector machine; LR, logistic regression; RF, random forest; XGBoost, Extreme Gradient Boosting; AUC, area under the curve.

**Figure 3:**
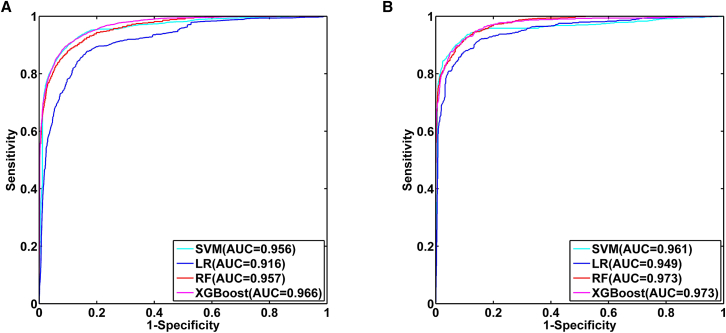
The ROC curves of IED detection for SVM, LR, RF, and XGBoost using 5-fold cross-validation (A) Full dataset: XGBoost shows the best performance with an AUC of 0.966 (95% CI, 0.962–0.970). (B) Daytime dataset: RF and XGBoost both exhibit superior performance with an AUC of 0.973 for each. ROC, receiver operating characteristic; IED, interictal epileptiform discharges; SVM, support vector machine; LR, logistic regression; RF, random forest; XGBoost, Extreme Gradient Boosting; AUC, area under the curve; CI, confidence interval.

We conducted an analysis on the daytime dataset to verify whether the models’ performance improved after excluding the effects of sleep-related confounding and circadian variations. The 5-fold cross-validation results of IED detection for the daytime dataset are presented in Table 4. In summary, all classifiers demonstrated better performance compared to that obtained from the full dataset, with AUC ≥0.940 (range: 0.949–0.973). Nevertheless, LR continued to underperform across all metrics. Notably, SVM achieved the highest specificity (95.4%; 95% CI, 94.3%–96.3%), accuracy (92.3%; 95% CI, 91.2%–93.3%), precision (91.5%; 95% CI, 89.3%–93.3%), and F1-score (0.892; 95% CI, 0.877–0.907). XGBoost maintained the best performance in sensitivity (87.1%; 95% CI, 84.8%–88.9%), F1-score (0.892; 95% CI, 0.875–0.905), and matched RF for the highest AUC (0.973; 95% CI, 0.967–0.978). Figure 2 shows the IED detection performance for the full and daytime datasets, while Figure 3 presents the corresponding receiver operating characteristic (ROC) curves.

**Table 4:** IED detection performance for different ML methods using 5-fold cross-validation for the daytime dataset

Method	Sensitivity (%)	Specificity (%)	AUC (--)	Accuracy (%)	Precision (%)	F1-score (--)
SVM	86.9 (84.6, 89.1)	95.4 (94.3, 96.3)	0.961 (0.952, 0.969)	92.3 (91.2, 93.3)	91.5 (89.3, 93.3)	0.892 (0.877, 0.907)
LR	83.3 (81.0, 85.6)	93.6 (92.3, 94.7)	0.949 (0.939, 0.957)	89.8 (88.6, 91.0)	88.3 (86.3, 90.4)	0.857 (0.840, 0.874)
RF	86.6 (84.4, 88.8)	95.1 (94.0, 961)	0.973 (0.967, 0.978)	92.1 (91.0, 93.1)	91.0 (89.2, 92.6)	0.888 (0.869, 0.901)
XGBoost	87.1 (84.8, 88.9)	95.2 (94.0, 96.2)	0.973 (0.967, 0.978)	92.2 (91.2, 93.3)	91.3 (89.5, 92.8)	0.892 (0.875, 0.905)

The data are reported as mean and 95% confidence interval.

RF, random forest; XGBoost, Extreme Gradient Boosting; CI, confidence interval.

### Model generalization ability

**Figure 4:**
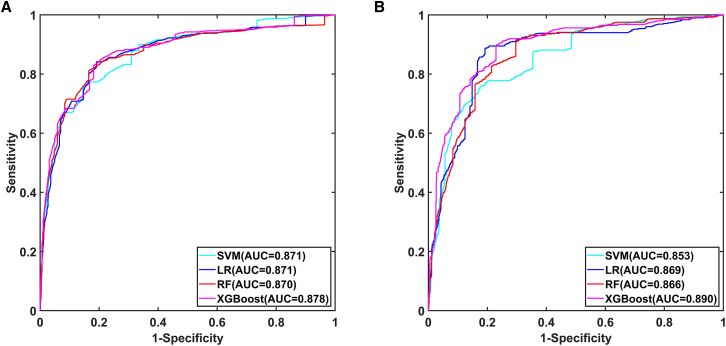
The ROC curves of IED detection for SVM, LR, RF, and XGBoost using patient-wise 5-fold cross-validation (A) Full dataset: XGBoost shows the best performance with an AUC of 0.878 (95% CI, 0.867–0.886). (B) Daytime dataset: XGBoost shows the best performance with an AUC of 0.890 (95% CI, 0.876–0.902). ROC, receiver operating characteristic; IED, interictal epileptiform discharges; SVM, support vector machine; LR, logistic regression; RF, random forest; XGBoost, Extreme Gradient Boosting; AUC, area under the curve; CI, confidence interval.

We randomly assigned all subjects in a 4:1 ratio to test the robustness and generalizability of the methods. All epochs from the same patient were used either for training or testing. The EEG dataset used for patient-wise 5-fold cross-validation is detailed in Table 5. The four ML models were retrained using patient-wise 5-fold cross-validation. The AUC was used to quantify the model’s generalization performance. The AUC values of these models, obtained through patient-wise 5-fold cross-validation for the full and daytime datasets, are shown in Figure 4. For the full dataset, XGBoost exhibited superior performance with an AUC of 0.878 (95% CI, 0.867–0.886). For the daytime dataset, XGBoost maintained the leadership with an AUC of 0.890 (95% CI, 0.876–0.902). Notably, AUC improvements were most pronounced for XGBoost compared to other algorithms. Up to this point, the XGBoost algorithm showed better performance than the other ML algorithms in terms of sensitivity and AUC. We, therefore, chose the XGBoost model as our final model.

**Table 5:** EEG dataset used for patient-wise 5-fold cross-validation

Dataset	Patient count	EEG label	Epochs (1 s)
One	28	IEDs	405
		Non-IEDs	719
Two	28	IEDs	547
		Non-IEDs	965
Three	28	IEDs	686
		Non-IEDs	1,156
Four	28	IEDs	391
		Non-IEDs	704
Five	29	IEDs	568
		Non-IEDs	1,089
Total	141	IEDs	2,597
		Non-IEDs	4,633

Beyond patient-wise cross-validation, we further assessed the model’s generalization to a completely different clinical population without epilepsy. The XGBoost model was evaluated on EEG recordings from 16 patients (9,069 one-second epochs), none of whom had a diagnosis of epilepsy or showed IEDs on clinical review. In this non-epilepsy control cohort, the model produced a false-positive rate of 28.46%, corresponding to a specificity of 71.54% in this population.

### Feature importance

**Figure 5:**
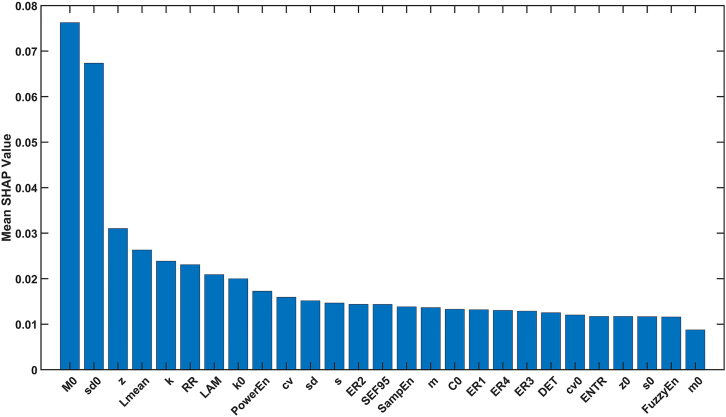
The mean importance of the 27 features

**Figure 6:**
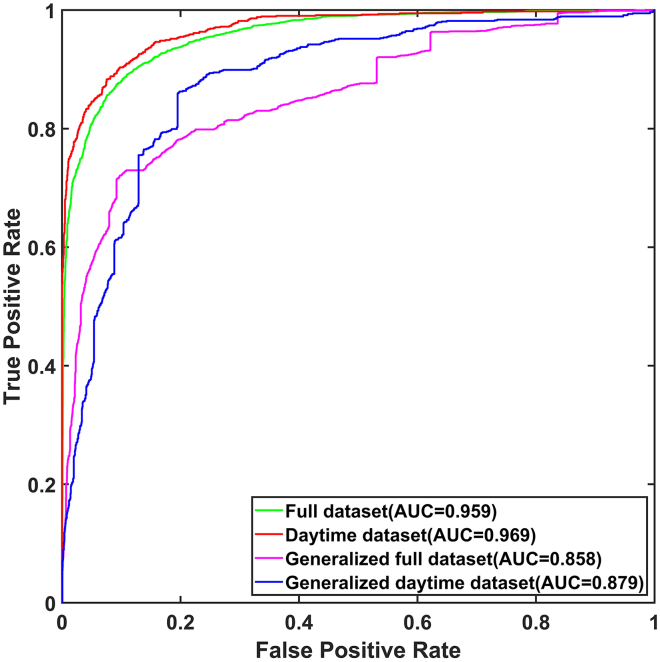
The ROC curves of IED detection for XGBoost based on the top 10 features using 5-fold cross-validation and patient-wise 5-fold cross-validation AUC values are 0.959 (95% CI, 0.955–0.964) and 0.969 (95% CI, 0.960–0.973) for the full and daytime datasets, respectively. For generalization performance, AUC values are 0.858 (95% CI, 0.848–0.866) and 0.879 (95% CI, 0.866–0.893) for the two datasets, respectively. ROC, receiver operating characteristic; IED, interictal epileptiform discharges; XGBoost, Extreme Gradient Boosting; AUC, area under the curve; CI, confidence interval.

The 27 features used in this study are listed in Table 6. Since XGBoost demonstrated the best overall performance among the four classifiers, we performed Shapley Additive Explanations (SHAP) analysis on the XGBoost model trained on the full dataset to identify the most important features for IED detection. The mean importance of the 27 features is displayed in Figure 5. We evaluated the classification performance of simplified versions of the XGBoost model containing the top 10 important features (i.e., M0, sd0, z, Lmean, k, RR, LAM, k0, PowerEn, and cv) on the full and daytime datasets. Figure 6 shows the 5-fold cross-validation and patient-wise 5-fold cross-validation results based on the top 10 important features for the full and daytime datasets, respectively. AUC values were 0.959 (95% CI, 0.955–0.964) and 0.969 (95% CI, 0.960–0.973) for the full dataset and daytime dataset, respectively. In the generalization analysis, the reduced model maintained acceptable performance, with an AUC of 0.858 (95% CI, 0.848–0.866) for the full dataset and an AUC of 0.879 (95% CI, 0.866–0.893) for the daytime dataset.

**Table 6:** List of all features

Method	Feature	Description
Time-domain analysis	m0, sd0, cv0, s0, k0, z0	The mean value, standard deviation, coefficient of variation, skewness, kurtosis, and zero-crossing rate
	M0, C0	Mobility and complexity of Hjorth parameters
	m, sd, cv, s, k, z	The mean value, standard deviation, coefficient of variation, skewness, kurtosis, and zero-crossing rate of cepstrum
Frequency-domain analysis	ER1, ER2, ER3, ER4	The ratios of energy in the four frequency bands of delta (0.5–4 Hz), theta (4–8 Hz), alpha (8–13 Hz), and beta (13–30 Hz) to the total power
	PowerEn	Power spectral entropy
	SEF95	95% spectral edge frequency
Nonlinear analysis	SampEn	Sample entropy
	FuzzyEn	Fuzzy entropy
	RR, DET, Lmean, ENTR, LAM	Recurrence rate, determinism, average diagonal line length, recurrence entropy, and laminarity of recurrence quantification analysis

### Exploratory analysis: Single-channel IED localization

**Figure 7:**
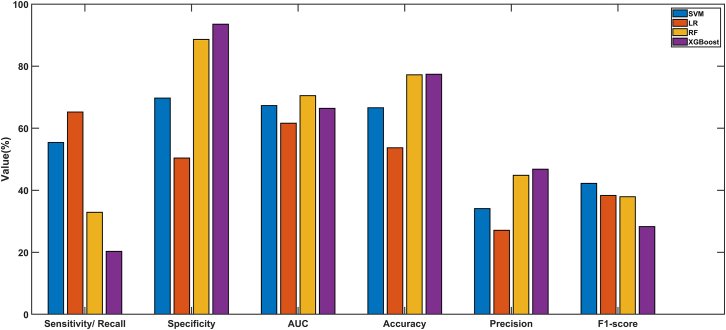
The performance of IED channel localization for the four ML methods RF achieved the highest AUC of 0.705. XGBoost showed high specificity of 93.5%. LR and SVM exhibited near-random performance (AUC = 0.616 and 0.673, respectively). IED, interictal epileptiform discharges; SVM, support vector machine; LR, logistic regression; RF, random forest; XGBoost, Extreme Gradient Boosting; AUC, area under the curve.

As an exploratory analysis separate from the primary IED detection task, we assessed the feasibility of single-channel IED localization using the proposed feature extraction strategy. To avoid information leakage, synthetic minority oversampling (SMOTE) was applied only to the training data within each cross-validation fold, with test data left untouched. Tenfold cross-validation was performed on the original, un-augmented dataset, and SMOTE was applied exclusively to balance the training set in each fold. The performance metrics were summarized in Figure 7. Under this unbiased evaluation protocol, all four classifiers showed limited performance for single-channel IED localization. RF achieved the highest AUC (0.705), but with low sensitivity (32.9%). XGBoost showed high specificity (93.5%) but very low sensitivity (20.3%). LR and SVM exhibited near-random performance (AUC = 0.616 and 0.673, respectively).

## Discussion

This study implemented four ML methods based on feature extraction to automatically detect IEDs. The performance of different ML classifiers was evaluated by performing 5-fold cross-validation on EEG dataset from 141 patients with epilepsy. The proposed Epilepsy-IEDs model using XGBoost achieved an excellent classification performance with a sensitivity of 84.6%, a specificity of 94.71%, an AUC of 0.966, an accuracy of 90.8%, a precision of 89.62%, and an F1-score of 0.868.

Beyond the larger cohort and superior metrics, this study offers several methodological contributions. First, we built a comprehensive feature space encompassing 27 time-domain, frequency-domain, and nonlinear features. Second, we systematically compared four ML algorithms (SVM, LR, RF, and XGBoost), with LR as a simple baseline, to identify the optimal classifier for IED detection. Third, we used both epoch-wise cross-validation and patient-wise hold-out validation to rigorously assess generalization performance. Fourth, we employed SHAP analysis to interpret model predictions and identify the most important features. These enhancements distinguish our work from previous studies and provide a reproducible framework for future IED detection research. The current study included a 141-patient cohort, which represents a 7- to 20-fold increase over previous studies.^11^^,^^13^^,^^15^^,^^16^ This larger sample size enhanced the statistical power and generalizability of the results, making the study’s conclusions more compelling. Unlike studies that relied on single-method approach,^12^^,^^13^^,^^15^^,^^17^ we compared multiple ML algorithms (SVM, RF, LR, and XGBoost) and ensured the identification of XGBoost as the optimal model through performance benchmarking. In terms of performance metrics, the AUC value of this study reached 0.967, significantly higher than Thomas et al.^18^ with 0.953 and Thomas et al.^20^ with 0.870, indicating that the Epilepsy-IEDs model had higher accuracy in distinguishing between positive and negative samples. The sensitivity (84.48%) and specificity (94.71%) of Epilepsy-IEDs were superior to those reported in previous studies, which typically ranged from 62% to 83% for sensitivity^13^^,^^21^^,^^23^ and from 63% to 78% for specificity.^12^^,^^19^ The accuracy (90.73%) and F1-score (0.867) of this study were also at a high level. Although the sensitivity of Epilepsy-IEDs was slightly lower than that reported in the literature,^14^^,^^15^^,^^16^^,^^19^^,^^22^ the number of patients in those studies was much smaller than ours, except for the study conducted by Sabor et al.^22^ Additionally, the specificity (94.71%) and precision (89.62%) of Epilepsy-IEDs were superior to most existing studies, demonstrating significant advantages in reducing false-positive rates. Our model not only effectively detected IED events but also reduced unnecessary alarms in clinical applications, thereby improving diagnostic reliability.

After excluding nighttime EEG recordings, the performance metrics of all ML classifiers improved. This improvement can be attributed to the superior signal quality of daytime recordings compared to nighttime recordings. Nighttime EEG is contaminated by sleep-related patterns (e.g., slow waves, spindles, and K-complexes), which resemble IEDs in morphology,^9^ and stage-specific oscillations (e.g., delta waves) overlap with IEDs in both time and frequency domains.^3^ Although we applied independent component analysis and artifact rejection to minimize these interferences, such waveforms are difficult to fully separate from genuine epileptiform discharges, leading to increased false-positives. In contrast, the background EEG during wakefulness is relatively clean due to effective artifact processing, and IED waveforms exhibit more stereotyped and consistent morphology, facilitating the model’s learning of discriminative features. However, daytime recordings also present unique challenges, such as movement-related artifacts (e.g., eye blinks and jaw clenching), which may introduce spike-like transients, and previous studies have shown that many patients exhibit higher IED rates during sleep compared to wakefulness. Nevertheless, all classifiers achieved higher performance on the daytime dataset, suggesting that the adverse effects of sleep-related physiological activities on IED detection outweigh the challenges posed by daytime movement artifacts and reduced IED frequency. Notably, the daytime dataset comprised approximately 35% of the total sample size (2,541 vs. 7,230 epochs), yet all classifiers consistently performed better on this smaller dataset, underscoring the importance of data quality over quantity in IED detection tasks.

It is crucial to ensure that the training and testing sets are independently partitioned. It was reported that the performance with an AUC of 0.93 on a patient-specific classifier would drop when applied to another patient, showcasing the need for external validation.^24^ Among reviewed works in Table 1, only a small portion met the requirement of independently separating training and testing sets while ensuring the number of patients.^17^^,^^20^^,^^21^^,^^22^ Although high detection performance was achieved, the generalization performance cannot be assessed due to the lack of external validation.^12^^,^^15^^,^^18^^,^^19^ In our study, we used patient-wise 5-fold cross-validation with 4:1 training-to-testing partitioning to assess out-of-sample performance. Despite expected AUC reductions (full dataset: 0.870–0.878; daytime dataset: 0.853–0.890), our models maintained clinically acceptable IED detection performance. Interestingly, when only considering daytime recordings, the AUC based on the XGBoost model was improved, while SVM, LR, and RF showed slight performance decreases. XGBoost consistently outperformed the other three methods in AUC, particularly on the daytime dataset. This superior performance is attributed to its gradient boosting mechanism, which iteratively learns from residuals to capture complex patterns. Its built-in L1 and L2 regularization prevents overfitting, crucial for smaller datasets.^25^ Additionally, XGBoost’s decision tree structure adapts well to complex features and noise in daytime dataset, maintaining stability even with limited data.^25^^,^^26^ In contrast, SVM is sensitive to feature distribution changes, LR struggles with nonlinear features, and RF may face overfitting and limited tree diversity in small-data scenarios, leading to their inferior performance. The consistent inferiority of LR across all evaluation metrics further highlights the value of employing more sophisticated modeling approaches. As a simple linear classifier, LR lacks the capacity to capture nonlinear relationships and feature interactions that characterize complex EEG patterns. While LR offers advantages in clinical interpretability and computational efficiency, its limited performance—particularly in sensitivity (77.5%) and AUC (0.916) on the full dataset—underscores the necessity of nonlinear decision boundaries and ensemble learning for accurate IED detection. The substantial performance gains achieved by XGBoost, RF, and SVM over LR thus validate the methodological choices made in this study. Consequently, we selected the XGBoost model as our ultimate model.

We further evaluated the XGBoost model on a non-epilepsy control cohort consisting of 16 patients without epilepsy (9,069 one-second epochs), which yielded a false-positive rate of 28.46% (i.e., a specificity of 71.54% in this non-epilepsy population). Encouragingly, despite being trained exclusively on epilepsy patients, the model correctly rejected more than 70% of non-IED epochs from a distinct non-epileptic population, indicating that its learned features retain a degree of discriminative capacity across different clinical populations. This relatively high false-positive rate may be explained by the presence of normal EEG variants that mimic IEDs in morphology, such as wicket spikes, rhythmic mid-temporal theta bursts, and 14- and 6-Hz positive bursts, which are known to occur more frequently in non-epileptic individuals than in epilepsy patients.^27^^,^^28^ These benign variants, along with physiological artifacts, can be misinterpreted by automated detectors as true epileptiform discharges.^28^ To our knowledge, no prior IED detection study has reported a false-positive rate specifically on a non-epilepsy cohort comprising diverse non-epileptic conditions. Most existing studies evaluate false-positives using non-IED segments from epilepsy patients rather than truly non-epileptic populations. For example, Thomas et al.^21^ reported a false detection rate of 0.2/min at 80% sensitivity across a mixed cohort, without isolating performance on non-epileptic individuals. While Saab et al.^29^ found that seizure detection models had significantly higher false-positive rates on non-epileptiform abnormalities (+19 percentage points), their task was seizure onset detection rather than IED detection. Therefore, our reported false-positive rate of 28.46% on a non-epilepsy cohort (including syncope, cerebral arteriosclerosis, dizziness, and other conditions) provides a clinically relevant benchmark for IED detector performance in a non-epilepsy population, addressing an important gap in the literature.

The SHAP method was employed to interpret XGBoost’s decision-making process. As expected, when the top 10 important features were included, the AUC values exceeded 0.95 for 5-fold cross-validation and 0.85 for patient-wise 5-fold cross-validation. The performance of the XGBoost model including only 10 features (Epilepsy-IEDs-10) closely approximated that of the full model. The SHAP method provides interpretable output from ML models, and experimental results demonstrated excellent IED detection performance with an AUC of 0.858–0.969 across all datasets. XGBoost showed a strong performance advantage with the simplified model containing only 10 features. As shown in Figure 6, time-domain features dominated the SHAP importance ranking. Hjorth mobility (M0) had the highest SHAP value, followed by standard deviation (sd0) and kurtosis (k0). M0 captures the average frequency of EEG signals; its elevation during IEDs reflects the high-frequency spikes and sharp waves characteristic of epileptic discharges, consistent with prior observations that IEDs exhibit increased energy in gamma-band frequencies.^30^ sd0 measures amplitude fluctuations; its high importance reflects the large-amplitude spikes associated with IEDs compared to relatively stable background activity. k0 describes signal peakedness, directly capturing the sharp, spike-like waveforms that define IEDs. Cepstrum-based features—zero-crossing rate (z), kurtosis (k), and coefficient of variation (cv)—further enhance detection by capturing signal sharpness, variability, and periodic patterns, which are particularly useful for identifying the abrupt and irregular waveforms of IEDs.^31^ In the frequency domain, power spectral entropy (PowerEn) was identified as a key feature, reflecting the increased spectral complexity and broader frequency distribution during IED events.^32^ Nonlinear features from recurrence quantification analysis—average diagonal length (Lmean), recurrence rate (RR), and laminarity (LAM)—were also crucial, as they quantify the periodicity, repetitiveness, and structural regularity of EEG signals, all of which are disrupted during epileptiform activity.^33^^,^^34^ Importantly, the top 10 features collectively capture the core electrographic signatures of IEDs from three complementary perspectives: spectral power distribution (M0 and PowerEn), amplitude-domain waveform morphology (sd0 and k0), and signal periodicity/regularity (cepstrum-based and RQA features). This alignment with established neurophysiological knowledge supports the clinical validity of our feature selection. Together, these features comprehensively represent the dynamic and complex nature of epileptic discharges, making them essential for accurate IED detection.

Our exploratory analysis of single-channel IED localization, conducted under a strict unbiased evaluation protocol (SMOTE applied only within each CV fold), yielded modest performance (best AUC = 0.705 for RF). Although RF’s ensemble learning via bootstrap sampling constructs diverse decision trees, making it highly robust to noise and outliers and enabling effective feature extraction from limited single-channel data,^35^ its performance remained modest in this task. These results indicate that accurate IED localization from single-channel EEG using handcrafted features alone is challenging. Several factors may explain this limitation: IEDs often involve multiple channels simultaneously, handcrafted features may not fully capture the complex spatiotemporal dynamics of IEDs, and class imbalance remains challenging even with SMOTE. In comparison, Thomas et al.^21^ achieved a channel localization sensitivity of 90.67% using a 1D CNN-based detector on a larger dataset (18,164 IEDs), suggesting that DL architectures may be more suitable for this task. However, their study did not report precision for channel localization, whereas our study provides comprehensive metrics including precision (46.8% for XGBoost), revealing that accurate channel localization with low false-positives remains challenging.

### Limitations of the study

This study has several limitations. First, the dataset was derived from a single center, and multi-center validation is needed to confirm the robustness of the findings. Second, although our cohort included 141 patients, the 2,597 annotated IEDs may not fully capture the morphological variability of IEDs. Larger and more diverse epilepsy datasets are required to improve model generalization. Third, our evaluation on a small non-epilepsy cohort of 16 patients yielded a false-positive rate of 28.46%, suggesting that the model may misinterpret normal EEG variants as IEDs. A larger and more diverse non-epilepsy control group is necessary to properly assess model specificity in real-world clinical settings where the pre-test probability of epilepsy is low. Fourth, although we collected sex information for all participants, we did not formally assess the influence of sex on model performance. Additionally, our exploratory single-channel IED localization analysis showed only modest performance (best AUC = 0.705), indicating that multi-channel approaches or DL methods may be more suitable for this task. Future work should expand both epilepsy and non-epilepsy datasets, incorporate DL architectures, and conduct systematic comparisons of ML algorithms to further advance IED detection.

## Resource availability

### Lead contact

Requests for further information and resources should be directed to and will be fulfilled by the lead contact, Hongyun Liu (ylooliu@163.com).

### Materials availability

This study did not generate new unique reagents.

### Data and code availability

• All data presented within this study are available in key resources table.
• All original code has been deposited at GitHub (https://github.com/ylooliu/Epilepsy-IEDs) and Zenodo (https://doi.org/10.5281/zenodo.20404907) and is publicly available as of the date of publication (see key resources table).
• Any additional information required to reanalyze the data reported in this work paper is available from the lead contact upon request.

## Acknowledgments

This study was supported by the 10.13039/501100012166National Key Research and Development Program of China under grant 2022YFC2405603.

## Author contributions

Conceptualization, R.A., P.Z., and G.W.; methodology, R.A., P.Z., H.L., and W.W.; investigation, R.A., P.Z., H.L., and W.W.; writing – original draft, R.A. and P.Z.; software, R.A. and P.Z.; validation, R.A. and P.Z.; formal analysis, R.A., P.Z., and G.W.; data curation, R.A., P.Z., and G.W.; writing – review & editing, G.W., H.L., and W.W.; supervision, G.W., H.L., and W.W.

## Declaration of interests

The authors declare that they have no known competing financial interests or personal relationships that could have appeared to influence the work reported in this article.

## STAR★Methods

### Key resources table

**Table:** 

REAGENT or RESOURCE	SOURCE	IDENTIFIER
**Deposited data**		

EEG dataset	This paper	https://github.com/ylooliu/Epilepsy-IEDs

**Software and algorithms**		

MATLAB R2024a	MathWorks	https://www.mathworks.com
EEG preprocessing	Swartz Center for Computational Neuroscience, University of California San Diego, CA, USA	https://sccn.ucsd.edu/eeglab
Feature extraction pipeline	This paper	https://github.com/ylooliu/Epilepsy-IEDs; https://doi.org/10.5281/zenodo.20404907
LIBSVM	C.C. & C.L. (National Taiwan University)	https://www.csie.ntu.edu.tw/∼cjlin/libsvm/
Logic regression	Andrew Ng (Stanford University)	https://github.com/trekhleb/machine-learning-octave
XGBoost	MATLAB Central File Exchange	https://www.mathworks.com/matlabcentral/fileexchange/75898-functions-to-run-xgboost-in-matlab?s_tid=srchtitle_support_results_1_xgboost
Random forest	Abhishek Jaiantilal (University of Colorado)	https://github.com/ajaiantilal/randomforest-matlab
SHAP	MATLAB implementation	https://www.mathworks.com
SMOTE	MATLAB Central File Exchange	https://github.com/dkbsl/matlab_smote

### Experimental model and study participant details

This study was approved by the Ethics Committee of Chinese PLA General Hospital (Approval No.: S2021-417-01). All participants provided written informed consent prior to enrollment. The study was conducted in accordance with the ethical standards set forth in the Declaration of Helsinki. We conducted an analysis of 24-h scalp EEG recordings from 155 patients with epilepsy. These data were captured during clinical evaluations at Chinese PLA General Hospital, adhering to the International 10–20 system for electrode placement. EEG recordings were consecutively collected from adult patients (age ≥18 years) with a confirmed diagnosis of focal epilepsy. Exclusion criteria were: pregnancy; substance abuse; severe systemic diseases (e.g., hypertension, diabetes, severe cardiopulmonary dysfunction, neurological disorders, thyroid dysfunction, etc.); recent use of sleep aids; inability to cooperate with the recording; poor EEG signal quality (e.g., excessive muscle artifact, electrode pop, etc.); presence of severe structural brain lesions; or incomplete clinical information. Each patient contributed at least one qualified EEG recording. We obtained a total of 141 IED EEG recordings, including 82 males (aged 37.7 ± 17.93 years) and 59 females (aged 40.5 ± 17.89 years), all participants were of Han Chinese ethnicity, recorded at a sampling frequency of 256 Hz or 512 Hz. IEDs were meticulously annotated by two independent neurologists, ensuring the accuracy and reliability of the annotations.

To assess the model’s specificity in a non-epileptic population, we additionally recruited a non-epilepsy control cohort of 16 patients who underwent EEG evaluations at the same center. These patients had no clinical diagnosis of epilepsy and no IEDs on their EEG recordings as confirmed by two independent neurologists. Their clinical diagnoses included cerebral arteriosclerosis (*n* = 4, 25.0%), syncope (*n* = 4, 25.0%), dizziness (*n* = 3, 18.8%), sleep disturbance (*n* = 2, 12.5%), orthostatic hypotension (*n* = 2, 12.5%), and dystonia (*n* = 1, 6.3%). This cohort comprised 8 males (aged 45.00 ± 10.79 years) and 8 females (aged 39.37 ± 9.87 years), all of Han Chinese ethnicity. The entire non-epilepsy cohort (16 patients, 9069 1-s epochs) was used to evaluate the model’s false positive rate, which yielded a value of 28.46%. Demographic and clinical characteristics of all participants are summarized in Table S1.

### Method details

A schematic overview of the complete analysis pipeline is provided in Figure 1. First, baseline EEG recordings from 155 patients were screened, yielding 141 qualified recordings. Two datasets were constructed for the primary IED detection task: the full dataset (*n* = 141, 2597 annotated IEDs and 4633 non-IEDs) and the daytime dataset (*n* = 55, 932 IEDs and 1609 non-IEDs). Second, a total of 27 features were extracted from three domains: time-domain, frequency-domain, and nonlinear analysis. Third, four ML classifiers (SVM, LR, RF, XGBoost) were trained using 5-fold cross-validation and the best-performing model was selected as Epilepsy-IEDs. Fourth, to test the models’ generalization ability, all subjects were randomly split into a 4:1 training-to-test set ratio. The models were then retrained using patient-wise 5-fold cross-validation for both full and daytime datasets. Additionally, to assess the model’s specificity in a non-epileptic population, we evaluated the XGBoost model on an independent non-epilepsy control cohort consisting of 16 patients without epilepsy (9069 1-s epochs). Fifth, SHapley Additive exPlanations (SHAP) analysis was performed on the Epilepsy-IEDs to identify feature importance, and a simplified model containing the top 10 important features (Epilepsy-IEDs-10) was constructed and validated. Finally, as an exploratory analysis, we examined the localization of IED channels based on single-channel EEG to assess the feasibility of the proposed feature extraction strategy for scalp IED localization. The synthetic minority oversampling (SMOTE) was applied to balance the dataset, followed by 10-fold cross-validation using the four ML classifiers. This analysis was performed as an additional investigation and was not part of the primary IED detection task.

#### Data preprocessing and segmentation

Given the transient nature of IEDs (20–200 ms duration),^36^ each IED epoch was extracted as a 1-s segment centered on the annotated event. To ensure the balance between the two classes of data, for each 24-h EEG recording, 1 to 100 segments of non-IED recordings were randomly selected, with each segment ranging from 1 to 20 s in length. Each non-IED record was then divided into 1-s epochs with 50% overlap. Thus, a total of 4633 non-IED epochs and 2597 IED epochs (along with the channel labels of occurrence) from 141 patients were obtained. Each epoch was a matrix of 19 × 512 (number of channels × time points). All epoch matrices were saved along with the expert annotations. To standardize the data, the EEG signals were resampled to 512 Hz. EEG data preprocessing was performed manually using EEGLAB 2021.0 (Delorme and Makeig, 2004) running under MATLAB R2024a (MathWorks, Natick, MA, USA). The EEG data were filtered within the range of 0.1–70 Hz and also subjected to a 50 Hz notch filter to address line noise. Subsequently, the EEG signals were re-referenced to the two ear electrodes (A1 and A2). Ear referencing was primarily chosen for its straightforward interpretation of EEG amplitude, frequency, and symmetry, as well as its robustness to movement artifacts. However, this montage may be suboptimal for detecting true temporal IEDs due to the risk of reference contamination. To mitigate this potential limitation, all IED candidates identified by the algorithm were secondarily reviewed and validated by two independent neurologists using bipolar and average montages, thereby excluding any instances of reference contamination. To minimize the impact of artifacts, the EEG signals underwent independent component analysis and artifact rejection processes to eliminate ocular and other artifactual interferences. The daytime subgroup (recordings taken between 8 a.m. and 5 p.m.) contains 55 patients with 1609 non-IED epochs and 932 IED epochs by excluding the nighttime recordings and ensuring that each patient has both non-IED and IED epochs. For the non-epilepsy control cohort, EEG recordings from 16 patients without epilepsy were processed using the same preprocessing pipeline. The duration of each EEG recording ranged from 110 to 961 s. From these recordings, a total of 9069 1-s non-IED epochs were extracted using a non-overlapping sliding window (i.e., consecutive 1-s segments without overlap). All epochs were confirmed to be free of IEDs by two independent neurologists.

#### Feature extraction

A total of 27 features were extracted from three complementary domains: time-domain statistics, frequency-domain spectral analysis, and nonlinear dynamic analysis, as detailed in Table 6. Time-domain features capture amplitude fluctuations and waveform morphology that directly reflect the sharp, high-amplitude nature of IEDs. Frequency-domain features characterize the spectral distribution shifts associated with epileptiform activity, particularly the increased energy in higher frequency bands. Nonlinear features quantify the complex, irregular dynamics of EEG signals during IED events, which may not be adequately captured by linear methods alone. This combination of linear and nonlinear features provides a comprehensive representation of EEG signals across multiple analytical perspectives, thereby maximizing the discriminative power of subsequent classifiers. Features from each channel were computed and then averaged to serve as input to the classifiers.

Time domain analysis of EEG focuses on directly extracting features from EEG signals such as mean value, standard deviation, zero-crossing rate, and so on. These time-domain features are valued because they are easy to calculate and can reflect the characteristics of EEG signals. In the detection of epilepsy, time-domain features are widely used due to its simplicity and adaptability.^37^ The Hjorth parameters, including activity, mobility and complexity, are commonly used to detect characteristics for the study of EEG signals.^30^ Mobility estimates the average frequency of the signal, complexity estimates the signal’s bandwidth by calculating the mobility of the first derivative of the EEG signal relative to the EEG signal itself. These parameters have application value in epilepsy detection, can provide important information about the EEG signal, and are helpful for identifying and analyzing epileptic seizures.^38^ Cepstrum refers to the inverse Fourier transform of the logarithm of the power spectrum. It can be used to capture periodic or repetitive patterns in EEG signals.^31^ The mean value, standard deviation, coefficient of variation, skewness, kurtosis, and zero-crossing rate of cepstrum were extracted.

Power spectral density of EEG was estimated using an auto-regression (AR) parametric model with order 14. Power ratios of four canonical frequency bands— delta (0.5–4 Hz), theta (4–8 Hz), alpha (8–13 Hz) and beta (13–30 Hz) —were computed relative to total spectral power. Power spectral entropy (PowerEn) is a kind of information entropy that aims to quantify the spectral complexity in frequency domain from an energy perspective.^32^ The 95% spectral edge frequency (SEF95) is a parameter that describes the power spectrum distribution of EEG signals. It refers to the frequency value at which the cumulative power spectrum of the signal reaches 95%. This parameter can reflect the overall frequency range of brain activity and is usually related to the depth of anesthesia and the state of brain function.^39^

Nonlinear features were derived to characterize the deterministic chaos and complex dynamics inherent in epileptiform activity. Sample entropy (SampEn) is a modified version of approximate entropy. It examines the similarity of different signal epochs and attributes high value to irregular and random signals.^40^ Fuzzy entropy (FuzzyEn) is a nonlinear index used to evaluate the probability of newly generated modes.^41^ FuzzyEn has been proven to be an outstanding index for detecting epileptic seizures effectively.^42^ Studies have shown that recurrence quantification analysis (RQA) can reveal dynamic differences in EEG signals between healthy and epileptic patients.^33^^,^^34^ The parameters of RQA such as recurrence rate (RR), determinism (DET), average diagonal line length (Lmean), entropy (ENTR) and lamination (LAM) can reflect these nonlinear dynamic characteristics of EEG signals.

#### Machine learning

We used four ML algorithms to develop the models: SVM, LR, RF and XGBoost. The four ML algorithms were selected based on their complementary strengths and widespread use in EEG-based epileptiform detection. SVM was chosen for its effectiveness in high-dimensional feature spaces and its ability to find globally optimal decision boundaries via kernel methods. LR was included as a simple, linear classifier to serve as a baseline. RF was selected for its robustness to noise and its ensemble learning mechanism that reduces overfitting. XGBoost was included due to its state-of-the-art performance in handling imbalanced datasets, its built-in regularization to prevent overfitting, and its capacity to capture complex nonlinear relationships through gradient boosting. Prior to model training, features were standardized across all samples to have a mean of 0 and a standard deviation of 1 to ensure scale invariance.

SVM is a powerful supervised learning model, widely used for the detection of IEDs.^11^^,^^12^^,^^17^^,^^19^^,^^20^ It seeks to find the optimal dividing hyperplane in the feature space that distinguishes different categories. The solution to SVM is a convex optimization problem, which ensures the discovery of a global optimum. SVM employs kernel functions to address nonlinear issues, mapping data into a higher-dimensional space to find the possibility of a linear separation.^19^ The kernel function used here is the radial basis function kernel. The two parameters C and γ were determined based on the optimal accuracy after 5-fold cross-validation and grid-search.^43^ In this work, the range for C was set from −8 to 8 and the range for γ was set from −4 to 4, with a step size of 0.8 for both. Thus, we obtained the optimal C as 9.1896 and the optimal γ as 0.0625.

Previous studies have shown that LR has application value in the detection of epilepsy.^44^^,^^45^ LR predicts the likelihood of an event by estimating probabilities and uses the sigmoid function to map the output of linear regression to a range between 0 and 1 for classification purposes. Here, log loss function was calculated as the cost function of LR and the gradient descent algorithm was used to update the model parameters. We set the classification probability threshold at 0.5.

RF is an ensemble-based learning technique used for classification.^35^ It consists of many individual decision trees, each derived from a separate bootstrap sample of the dataset, with each tree classifying the data. The final outcome is determined by a majority vote among the trees. RF has established efficacy in both epilepsy and IED detection.^13^^,^^15^^,^^19^

XGBoost is a state-of-the-art efficient gradient-boosted decision tree algorithm. It has been improved based on the traditional gradient boosting decision tree, enhancing the model’s performance and training speed.^25^ The core of XGBoost is the use of the boosting concept, which integrates multiple weak learners (usually decision trees) into a strong learner, achieving joint decision-making of multiple trees. This forward-adding model uses the result of each tree as the difference between the target value and the predicted results of all previous trees, and then sums up all the results to get the final predicted value, thereby improving the model’s effectiveness. Studies have shown that XGBoost can predict and detect epilepsy by analyzing EEG signals.^46^

#### Model explanation

To interpret feature importance, SHAP analysis was performed on the Epilepsy-IEDs. We used the SHAP algorithm to explain the model’s feature importance and to construct a simplified model for clinical use. SHAP is a game-theoretic approach that assigns a value to each feature, representing its marginal contribution to the model’s prediction.^47^ Unlike traditional feature importance methods (e.g., Gini importance or permutation importance), SHAP provides both global and local interpretability by calculating the marginal contribution of each feature for every individual prediction. Additionally, SHAP is model-agnostic and can be applied to any machine learning model without modifying the underlying algorithm, and its additive property ensures that the sum of SHAP values for all features equals the difference between the model’s prediction and the expected baseline value. SHAP provides local explanations for model predictions by calculating the average marginal contribution of each feature across all possible feature combinations. SHAP values are applicable to any ML model, including complex black-box models, and can be visualized using bar charts or force plots to intuitively display feature contributions. A positive SHAP value indicates that a feature increases the probability of predicting the positive class, while a negative value indicates the opposite.

#### Data augmentation

For IED channel localization (the exploratory analysis based on single-channel EEG), a total of 10867 IED channel labels and 38476 non-IED channel labels were manually annotated by the clinical experts. To address class imbalance between IED and non-IED samples in this specific analysis, SMOTE was applied only to the training data within each cross-validation fold. Specifically, 10-fold cross-validation was performed on the original, un-augmented dataset. For each fold, the training set was balanced using SMOTE (k-nearest neighbors = 5, sampling ratio = 2.5), while the corresponding validation set remained as untouched, original data for unbiased performance evaluation. This process was repeated across all 10-folds, and the final performance metrics were averaged. SMOTE generates synthetic samples for the minority class by interpolating between k-nearest neighbors in feature space, thereby balancing class distribution without introducing bias.^48^ It should be noted that SMOTE was used exclusively for this exploratory IED channel localization analysis and was not applied to the primary IED detection task.

### Quantification and statistical analysis

#### Evaluation metrics

We applied the same pipeline and a 5-fold cross-validation process to evaluate the performance of the classification methods. Accuracy alone does not provide sufficient information about the classifier’s performance and is not suitable for comparing different methods because it generally depends on the prior likelihood of an IED.^10^ Thus, we performed the ROC curves to assess the performance of the classifiers. In addition, precision and the F1-score are also used as reference metrics for classification performance. Classification performance across all algorithms is presented in Table 2. True positive (TP) refers to the number of true IEDs predicted as IEDs. False negative (FN) refers to the number of true IEDs predicted as non-IEDs. True negative (TN) refers to the number of true non-IEDs predicted as non-IEDs. False positive (FP) refers to the number of non-IEDs predicted as IEDs.

We used bootstrapping with 500 resamples to acquire the mean value and the 95% CI of the performance metrics (i.e., sensitivity, specificity, AUC, accuracy, precision, and F1-score). All statistical analysis and EEG data processing were performed using MATLAB R2024a (The MathWorks, Inc., Natick, MA).
