# Supplementary material for: Epilepsy-IEDs: An automated machine learning model for detecting interictal epileptiform discharges from scalp electroencephalograms
Source: iScience. 2026 Jun 22;29(7):116408. doi: 10.1016/j.isci.2026.116408 (PMC13316274; doi:10.1016/j.isci.2026.116408)
Supplement: Document S1. Table S1 [file mmc1.pdf]

iScience, Volume *29*

Supplemental information

Epilepsy-IEDs: An automated machine learning model for detecting interictal epileptiform discharges from scalp electroencephalograms

Ran Ao, Ping Zhan, Guojing Wang, Hongyun Liu, and Weidong Wang

Table S1. Demographic and clinical characteristics of participants, related to STAR Methods.

Characteristic	Epilepsy patients (n=141)	Non-epilepsy patients (n=16)
Sex, n (%)		
Male	82 (58.2%)	8 (50.0%)
Female	59 (41.8%)	8 (50.0%)
Age (years, mean ± SD)		
Overall	38.89 ± 17.91	42.19 ± 13.87
Male	37.7 ± 17.93	45.00 ± 10.79
Female	40.5 ± 17.89	39.37 ± 9.87
Ethnicity, n (%)		
Han Chinese	141 (100%)	16 (100%)
Clinical diagnosis, n (%)		
Epilepsy	141 (100%)	-
Cerebral arteriosclerosis	-	4 (25.0%)
Syncope	-	4 (25.0%)
Dizziness	-	3 (18.8%)
Sleep disturbance	-	2 (12.5%)
Orthostatic hypotension	-	2 (12.5%)
Dystonia	-	1 (6.3%)
